# Neurodegeneration in the centrally-projecting Edinger–Westphal nucleus contributes to the non-motor symptoms of Parkinson’s disease in the rat

**DOI:** 10.1186/s12974-022-02399-w

**Published:** 2022-02-02

**Authors:** Balázs Ujvári, Bence Pytel, Zsombor Márton, Máté Bognár, László Ákos Kovács, József Farkas, Tamás Gaszner, Gergely Berta, Angéla Kecskés, Viktória Kormos, Boglárka Farkas, Nóra Füredi, Balázs Gaszner

**Affiliations:** 1 Department of Anatomy, Research Group for Mood Disorders, Medical School, University of Pécs, Szigeti út 12., 7624 Pecs, Hungary; 2 Department of Medical Biology, Medical School, University of Pécs, 7624 Pecs, Hungary; 3 Department of Pharmacology and Pharmacotherapy, Medical School & Szentágothai Research Centre, Molecular Pharmacology Research Group, University of Pécs, 7624 Pecs, Hungary; 4 Centre for Neuroscience, University of Pécs, 7624 Pecs, Hungary

**Keywords:** Rotenone, Saporin, Urocortin 1, Depression, Anxiety, Rat, RNAscope, Immunofluorescence

## Abstract

**Background:**

The neuropathological background of major depression and anxiety as non-motor symptoms of Parkinson’s disease is much less understood than classical motor symptoms. Although, neurodegeneration of the Edinger–Westphal nucleus in human Parkinson’s disease is a known phenomenon, its possible significance in mood status has never been elucidated. In this work we aimed at investigating whether neuron loss and alpha-synuclein accumulation in the urocortin 1 containing (UCN1) cells of the centrally-projecting Edinger–Westphal (EWcp) nucleus is associated with anxiety and depression-like state in the rat.

**Methods:**

Systemic chronic rotenone administration as well as targeted leptin–saporin-induced lesions of EWcp/UCN1 neurons were conducted. Rotarod, open field and sucrose preference tests were performed to assess motor performance and mood status. Multiple immunofluorescence combined with RNAscope were used to reveal the functional–morphological changes. Two-sample Student’s *t* test, Spearman’s rank correlation analysis and Mann–Whitney *U* tests were used for statistics.

**Results:**

In the rotenone model, besides motor deficit, an anxious and depression-like phenotype was detected. Well-comparable neuron loss, cytoplasmic alpha-synuclein accumulation as well as astro- and microglial activation were observed both in the substantia nigra pars compacta and EWcp. Occasionally, UCN1-immunoreactive neuronal debris was observed in phagocytotic microglia. UCN1 peptide content of viable EWcp cells correlated with dopaminergic substantia nigra cell count. Importantly, other mood status-related dopaminergic (ventral tegmental area), serotonergic (dorsal and median raphe) and noradrenergic (locus ceruleus and A5 area) brainstem centers did not show remarkable morphological changes. Targeted partial selective EWcp/UCN1 neuron ablation induced similar mood status without motor symptoms.

**Conclusions:**

Our findings collectively suggest that neurodegeneration of urocortinergic EWcp contributes to the mood-related non-motor symptoms in toxic models of Parkinson’s disease in the rat.

**Supplementary Information:**

The online version contains supplementary material available at 10.1186/s12974-022-02399-w.

## Background

Parkinson's disease (PD) is characterized by classic motor symptoms including rigor, tremor and bradykinesia attributed to neurodegeneration in the substantia nigra pars compacta (SNpc) and consequent loss of striatal dopaminergic afferentation [1]. According to the self-assessment of patients, non-motor symptoms deteriorate their quality-of-life more dramatically than motor symptoms themselves [2]. Therefore, the attention has been recently turning to PD-associated non-motor symptoms, such as anxiety and depression. While the main neuropathological hallmarks of motor symptoms such as Lewy body (LB) and Lewy neurite accumulation as well as nigral neurodegeneration are relatively well-investigated [1], much less knowledge has collected explaining the non-motor psychiatric symptoms. Anxiety disorders as well as major depression have been linked with altered noradrenergic, serotonergic and dopaminergic neurotransmission [3], but the underlying neuropathology of PD-associated mood disorders is poorly understood. Not surprisingly, the currently available pharmacotherapeutic strategies sharing the goal to increase serotonin (5-HT) and/or norepinephrine levels in the brain, provide limited efficacy [4].

Some neuropathological observations may explain the association between PD and mood disorders. For instance, LBs accumulate in the serotoninergic raphe nuclei [3], dopaminergic ventral tegmental area (VTA) as well as in the norepinephrinergic locus ceruleus (LC) [5]. Besides these, neurodegeneration and LB accumulation have been observed in PD in the dorsal vagal nuclear complex and in the Edinger–Westphal nucleus (EW) [6, 7].

Classically, cholinergic parasympathetic preganglionic neurons of the EW project to the ciliary ganglion controlling the sphincter pupillae and ciliary muscles. Based on this, patients with PD would be expected to suffer from compromised pupillary light reflex and lens accommodation [8]. However, this is usually not the case, even though it is known that the "rostral part of the EW suffers 50% neural loss and 3% of the cells contain Lewy bodies" [6]. This paradox suggested us that the recently defined peptidergic centrally-projecting (EWcp) division [9] of the EW may be affected.

The EWcp expresses leptin receptors [10] and a number of neuropeptides [11–14] including urocortin 1 (UCN1) [15, 16]. UCN1 belongs to the corticotropin-releasing hormone (CRH) peptide family [15] and activates both CRH receptors. The significance of CRH receptor signaling has been assessed in numerous biological functions including stress adaptation response [17]. The involvement of UCN1 in stress and depression models has been demonstrated in mouse [18, 19], rat [20–23] and non-human primate [24] depression models. Importantly, EWcp samples of male depressed suicide victims contained decreased amount of *Ucn1* mRNA [25].

In this work our goal was to investigate the putative underlying mechanisms of mood disorders in PD. Our main hypothesis was that the specific loss or damage of EWcp/UCN1 neurons directly contributes to anxiety and depression as non-motor symptoms of PD.

To test this, a systemic chronic rotenone treatment model for PD was applied in the rat [26]. As EWcp/UCN1 neurons carry leptin receptors [10], a leptin-conjugated saporin-induced selective ablation of UCN1 neurons was also performed to validate the depression-like phenotype in rats. Behavioral, functional–morphological, biochemical and histopathological tools were used to test the motor coordination, mood status as well as morphological changes in the brain.

## Methods

### Animals

In-house bred male Wistar rats (breeding pairs purchased from Animalab Kft., Vác, Hungary) were housed in three-per-cage (40 × 25 × 20 cm) groups in temperature and humidity-controlled environment. Animals were supplied with standard rodent chow and tap water ad libitum. Experiments were approved by the Animal Welfare Committee of Pécs University, the National Scientific Ethical Committee on Animal Experimentation in Hungary and the National Food Chain Safety Office in Hungary (license No: BA02/2000-49/2017).

### Systemic rotenone treatment model of PD

Eleven-month-old animals were subjected to 6 weeks daily subcutaneous rotenone treatment [(R8875-1G, Sigma, Budapest, Hungary) (*n* = 23; 1.5 mg/kg/day rotenone dissolved in 20 µl/kg/day dimethyl-sulfoxide (Fisher Scientific, Loughborough, UK) and 1 ml/kg sunflower oil vehicle (8000-21-6, Molar Chemicals Kft., Halásztelek, Hungary)] vs. vehicle injected controls (*n* = 12). Five rats due to humane endpoint and six others showing less than 20% SNpc dopaminergic neuron loss upon rotenone treatment were removed from the study [27]. Finally, 12 rotenone-treated rats were included.

#### Sucrose preference test

The anhedonia level was determined by sucrose preference test (SPT). Seventy-two hours before testing 1 m/v% sucrose solution and tap water were offered. After 24 h, the place of the bottles was reversed. On the test day, water deprivation was applied between 6 a.m. and 4 p.m. Then, the two bottles were offered for individually caged animals for 3 h. Sucrose preference was calculated according to the widely used formula [28].

#### Open field test

The open field test (OFT) was performed in a brightly lit box [50 × 50 × 38 cm] with black background. Video recordings of 5 min were analyzed with SMART Junior Tracking software (PanLab, Barcelona, Spain) to assess the time spent in the periphery of the box, and the traveled distance [29].

#### Rotarod test

Rotarod performance test (RPT) was used to assess motor coordination. Animals were first pre-trained to run on the accelerating rotating axis three times. For testing, rats were placed on the axis of the device (47750, Ugo Basile, Gemonio, Italy) and the time interval rats spent on the rotating axis was registered [30].

### Targeted toxin-induced lesion of EWcp/UCN1 neurons

The EWcp contains neuron populations that do not express UCN1 [14, 31]. Because in this project, our aim was to study the significance of UCN1 neuron loss, we applied a selective neuron ablation by saporin. This neurotoxin enters neurons only if it is conjugated to a substance that is internalized by receptor-mediated endocytosis and irreversibly inhibits the cells’ protein synthesis [32]. As EWcp/UCN1 neurons carry leptin receptors [10], leptin-conjugated saporin injection provides a reliable tool to perform selective UCN1 neuron ablation [33].

Thirty-two rats were intraperitoneally anesthetized by ketamine (78 mg/kg, Richter, Budapest, Hungary) and xylazine (13 mg/kg, Eurovet, Nagyatád, Hungary). Using a stereotaxic apparatus, 0.08 μl leptin-conjugated saporin (n = 16) or saporin (*n* = 16) solution (#KIT-47, ATS INC, Carlsbad, CA, USA) was injected into the EWcp area. From a point 1.5 mm left and 4.8 mm caudal to the Bregma [34] an oblique injection path closing 19° angle with the vertical axis was set and the Hamilton needle was introduced to a depth of 5.8 mm [33, 35]. Compounds were injected in 30 s. Thirty seconds later, the needle was retracted by 0.5 mm, and 1 min later, it was removed.

If the magnitude of UCN1 neuron loss was less than 20% upon leptin–saporin treatment, the animal was excluded (8 rats) from the experiment. Six rats were excluded, because the injection path deviated from the planned route, or the needle caused physical damage in the EWcp. Finally, nine saporin (control) and leptin–saporin-injected rats were included into the statistics. Behavioral testing was performed after 10 days recovery as described above. Behavioral data were converted into *Z*-scores using the formula *Z* = (*X* − *µ*)/*σ*, where (*X*) represents the raw data of the individual animal, (*µ*) and (*σ*) the mean and standard deviation of the control group, respectively [36]. Tissue sampling was performed 2 weeks after surgery.

### Tissue and sample preparation

Intraperitoneally anesthetized (urethane, 1.4 g/kg, Merck KGaA, Darmstadt, Germany) rats were perfused with 50 ml 0.1 M phosphate-buffered saline (PBS, pH 7.4) followed by 250 ml 4% paraformaldehyde in Millonig’s buffer. Brains were dissected and post-fixed for 14 days. Thymus and adrenals were collected and weighed.

Thirty µm coronal vibratome (Leica Biosystems, Wetzlar, Germany) sections in six series were collected between the optic chiasm and middle cerebellar peduncle. Sections were stored in PBS containing 0.01% sodium azide at 4 °C [22].

### Multiple immunofluorescence labeling

Sections of caudate–putamen (CPu) [Bregma − 0.36 mm − (− 0.84 mm)], EWcp [Bregma − 5.88 mm – (− 6.72 mm)], SNpc [Bregma − 5.16 mm – (− 6.24 mm)], VTA [Bregma − 5.16 mm – (− 6.24 mm)], dorsal- (DR) [Bregma − 6.96 mm – (− 7.32 mm)] and median (MNR) raphe nucleus [Bregma − 7.20 mm – (− 7.80 mm)] LC and A5 noradrenergic cells [Bregma − 9.60 mm – (− 10.08 mm)] were manually selected [34] for multiple immunofluorescence labelings on samples from the rotenone model. After epitope retrieval (10 min, 90 °C, sodium–citrate buffer; pH 6.0), permeabilization (0.5% Triton X-100; Sigma) and 2% normal donkey serum (60 min) treatment, sections were incubated in cocktails of primary antibodies for double (16 h, room temperature) or triple labelings (48 h, 4 °C). Ionized calcium-binding adapter molecule 1 (IBA1, rabbit)-tyrosine-hydroxylase (TH, mouse) double labeling was performed in the SN, VTA, LC and A5. 5-HT (mouse)-IBA1 labeling was conducted in the DR and MNR. CPu was assessed by TH (rabbit)-NeuN (mouse) labeling. UCN1 (goat)–IBA1–glial fibrillary acidic protein (GFAP, mouse); moreover, UCN1 (goat)–alpha-synuclein (mouse)–TH (rabbit) triple stainings were performed. Reactive microglia were identified by IBA1 (goat)–CD68 (rabbit) double labeling in the EWcp. Tumor necrosis factor alpha (TNF-alpha, rabbit)–GFAP (mouse)–IBA1 (goat), moreover, inducible nitric oxide-synthase-(iNOS, rabbit)–GFAP (mouse)–IBA1 (goat) triple labelings were conducted in the SNpc. In the targeted toxin model, UCN1 (rabbit)–GFAP (mouse)–IBA1 (goat) and a UCN1 (goat)–TH (mouse)–caspase 3 (rabbit) triple labelings, moreover, a UCN1 (goat)–NeuN (mouse) double labeling was performed. The respective fluorophore-conjugated or biotinylated secondary sera were applied either for 3 h in double or overnight in triple stainings. Biotinylated donkey anti-rabbit serum was detected by Cy5-conjugated streptavidin (Jackson, 1:1500, 3 h). Finally, sections were mounted on gelatin-coated slides, air-dried and covered with glycerol-PBS.

Antisera were characterized earlier as referenced in Table 1. Omission and non-immune serum replacement of primary or secondary antibodies prevented all immunolabelings (images not shown).

**Table 1:** Summary of antibody details

Antibody name	Vendor	Cat#	References	RRID	Antigen	Dilution
Anti-tyrosine-hydroxylase (host: rabbit, polyclonal)	Abcam	ab112	[65]	AB_297840	Full length protein from rat pheochromocytoma	1:2000
Anti-tyrosine-hydroxylase (host: mouse, monoclonal)	Sigma	T2928	[66]	AB_477569	rat tyrosine-hydroxylase	1:2000
Anti-UCN1 (host: goat, polyclonal)	Santa Cruz	SC1825	[21]	AB_2304014	C terminus peptide fragment of rat UCN1	1:200
Anti-UCN1 (host: rabbit, polyclonal)	Salk Institute Prof. WW. Vale	PBL#5779	[67]	AB_2315527	rat UCN1 (amino acids 1–40)	1:20,000
Anti-GFAP (host: rabbit, monoclonal)	Abcam	Ab33922	[68]	AB_732571	C terminus synthetic peptide (sequence commercially sensitive)	1:1500
Anti-GFAP (mouse monoclonal)	Novocastra	NCLLGFAP-GA5	[69]	AB_563739	Porcine spinal cord	1:1000
Anti-IBA1 (host: rabbit, polyclonal)	Wako Ltd	019–19,741	[70]	AB_839504	C terminus synthetic peptide of IBA1	1:1000
Anti-IBA1 (host: goat, polyclonal)	Wako Ltd	011–27,991	[71]		Synthetic peptide corresponding to C-terminal of IBA1	1:500
Anti-alpha-synuclein (host: mouse, monoclonal)	Abcam	Ab1903	[72]	AB_302665	Recombinant full length protein corresponding to human alpha-synuclein	1:2000
Anti-NeuN (host: mouse, monoclonal, (clone A60)	Millipore	MAB377	[73]	AB_2298772	Purified cell nuclei from mouse brain	1:1000
Anti-5-HT (host: mouse, monoclonal)	Universite´ Claude Bernard, Lyon, France Dr. Luciene Leger	Custom made	[18]		BSA-conjugated 5HT	1:20,000
Anti-CD68 (Host: rabbit, polyclonal)	Abcam	ab125212	[74]	AB_10975465	Synthetic peptide AFCITRRRQSTYQPL	1:100
Anti-caspase 3 (host: rabbit, polyclonal)	Abcam	ab49822	[75]	AB_868673	aa167-175 within the p17 subunit (cleaved caspase)	1:4000
Anti-TNF-alpha (host: mouse, monoclonal)	Abcam	ab220210	[74]	AB_2892586	Recombinant full length protein corresponding to human TNFalpha	1:200
Anti-iNOS (host: rabbit, monoclonal)	Abcam	ab178945	[76]	AB_2861417	Recombinant fragment (information is proprietary to the supplier)	1:250
Normal Donkey Serum	Jackson Immunoresearch	017–000-121		AB_2337258	-	2%
Alexa Fluor 488 donkey anti-mouse IgG (H + L)	Jackson Immunoresearch	706–545-148		AB_2340472	-	1:400
Alexa Fluor 488 donkey anti-goat IgG (H + L)	Jackson Immunoresearch	706–545-148		AB_2340472	-	1:600
Cy3 donkey Anti-Rabbit IgG (H + L)	Jackson Immunoresearch	711–165-152		AB_2307443	-	1:800
Alexa Fluor 647 donkey Anti-Rabbit IgG (H + L)	Jackson Immunoresearch	711–605-152		AB_2492288	-	1:500
Alexa Fluor 647 donkey Anti-Goat IgG (H + L)	Jackson Immunoresearch	705–605-003		AB_2340436	-	1:500
Biotin-SP (long spacer) donkey anti-rabbit IgG (H + L)	Jackson Immunoresearch	711-065-152		AB_2340593	-	1:200

### RNAscope in situ hybridization combined with immunofluorescence

In the rotenone model, four EWcp sections per animal were pretreated for RNAscope according to our recently developed protocol [37] optimized for 30 µm-thick PFA fixed sections. Subsequent steps of RNAscope protocol was performed according to the supplier’s suggestions. *Ucn1* mRNA was visualized by Cy5 (1:3000). A mouse *Ucn1* mRNA probe (Cat No: 466261, Advanced Cell Diagnostics, Newark, CA, USA) was used as mouse and rat UCN1 show full peptide sequence identity [38]. Some randomly selected sections were hybridized with triplex positive control probes for the rat (320891) or with triplex negative control probes (320871). After channel development positive control gave well-recognizable signal, while no fluorescence was seen in negative controls (images not shown).

Subsequently, slides were further processed for double-immunofluorescence using goat anti-UCN1 (1:175) and rabbit IBA1 (1:1000) for 48 h at 4 °C. After washes, Alexa 488-conjugated donkey anti-goat and Cy3-conjugated donkey anti-rabbit sera (1:500, 3 h) were used. Sections were counterstained with 4′,6-diamidino-2-phenylindole (DAPI) and covered with antifade medium.

### Microscopy, digitalization and morphometry

Samples were digitalized using Olympus FluoView 1000 confocal microscope with × 20 (NA: 0.5), × 40 (NA: 0.8) and × 60 (NA: 1.49 oil) objectives. The excitation and emission of fluorophores were set according to the built-in settings of the FluoView software (Fv10-ASW; Version 0102). Blue (DAPI), red (Cy3), green (Alexa 488) and white (Cy5 and Alexa 647) virtual colors were used.

Morphometry including counting of cells, nerve terminals and signal dots of the RNA labeling, densitometry and cell size measurement was performed on non-edited digital images. Every quantitation was carried out on four to six images per animal. The intensity of fluorescent signal was semi-quantified by measurement of the cytoplasmic signal corrected for the background, yielding the specific signal density (SSD) [18]. The measurement and unbiased stereological cell counting [39] was performed by ImageJ software (version 1.52a, NIH). Glial cell morphology was conducted according to the scoring system by Harrison et al. [40]. For publication purposes, representative images were cropped, contrasted and edited using Adobe Photoshop software.

### Statistics

Data were presented as mean of the group ± standard error of the mean. Statistics were performed by two-sample Student’s *t* test after assessment of data distribution. Outlier data beyond the 2-sigma range were excluded. Spearman’s rank test was also applied to search for correlations. Mann–Whitney *U* test was used to assess results of glial activity scores. Alpha was set to 5% in all cases.

## Results

### Partial loss of EWcp/UCN1 neurons in the rotenone model is associated with depression-like phenotype and increased anxiety

**Fig. 1:**
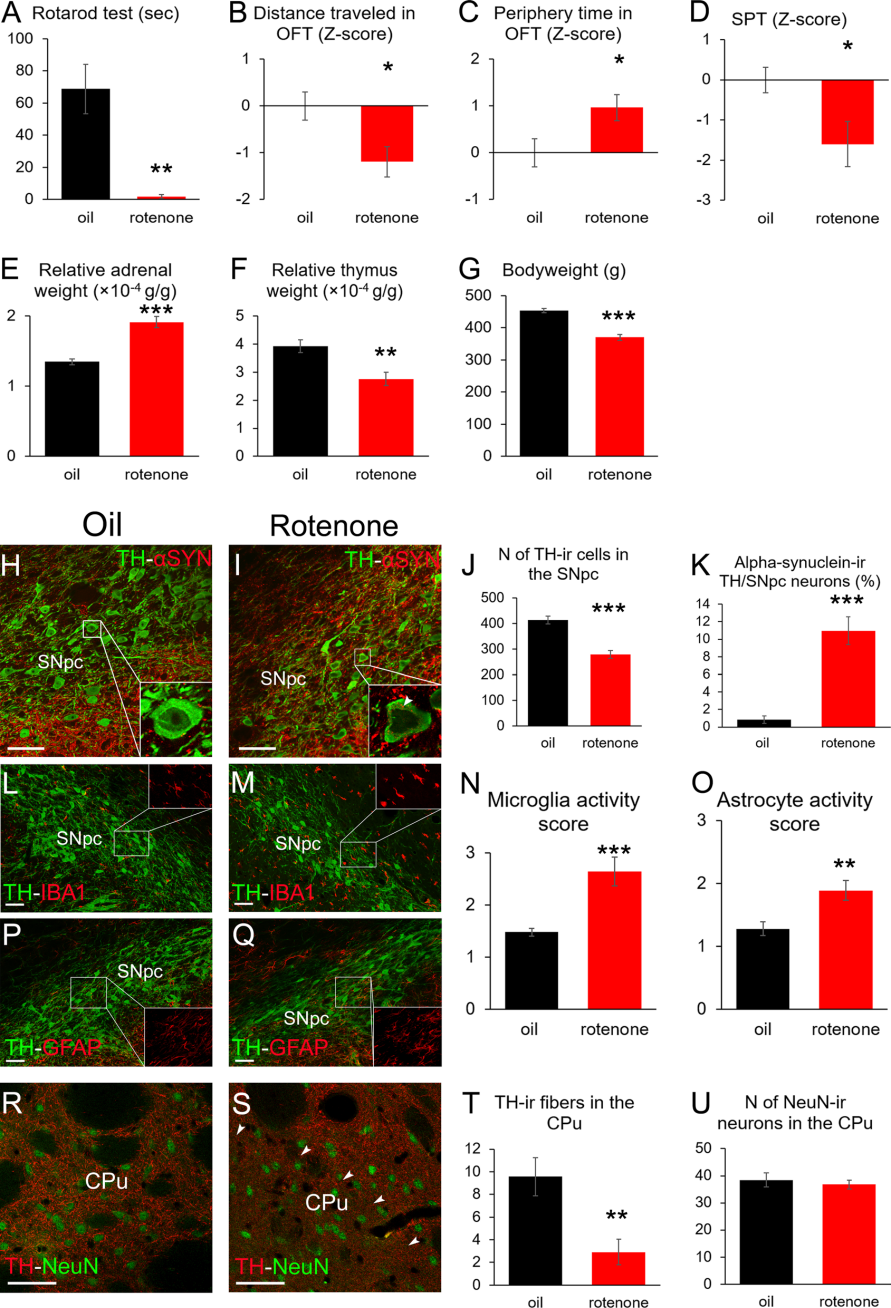
Increased anxiety and depression-like behavior in the rotenone model of Parkinson’s disease. Rotenone-treated rats (red bars) were unable to run on the rotating rod in the rotarod test (**A**) and they moved less in the open field test (OFT) (**B**). The increased anxiety level was proven by longer time spent in the periphery of the OFT arena (**C**). Rotenone-treated rats drank less sweetened water in the sucrose preference test (SPT) suggesting increased anhedonia (**D**). Relative adrenal (**E**) and thymus (**F**) weights as well as bodyweight (**G**) data suggest increased hypothalamus–pituitary–adrenal axis activity in the rotenone-treated group compared to vehicle (oil) injected rats (black bars). Tyrosine-hydroxylase (TH, green in **H**, **I**)—alpha-synuclein (αSYN, red in **H**, **I**) double labeling in the substantia nigra pars compacta (SNpc) revealed reduced SNpc/TH-immunoreactive (ir) cell count (**J**) in rotenone-treated rats. Cytoplasmic alpha-synuclein-ir inclusions (see arrowhead in insert of panel **I**) were observed upon rotenone treatment in SNpc/TH neurons (**K**). The assessment of microglia morphology in the SNpc by TH (green in **L**, **M**)—ionized calcium binding adaptor molecule 1 (IBA1, red in **L**, **M**) double labeling revealed increased activity score upon rotenone treatment (**N**). TH (green in **P**, **Q**)—glial fibrillary acidic protein (GFAP, red in **P**, **Q**) double labeling revealed also increased astrogliosis (**O**) in rotenone-treated rats. TH (red in **R**, **S**)—NeuN (green, **R**, **S**) labeling in the caudate-putamen (CPu) revealed reduced dopaminergic fiber density in rotenone-treated rats (**T**) occasionally with focal pattern (see the border of focal fiber loss area indicated by arrowheads in panel **S**). Rotenone treatment did not induce neuron loss in the striatum (**U**). *p < 0.05, **p < 0.01, ***p < 0.001 in Student’s *t* test or in Mann–Whitney *U* test in **N** and **O**. *n* = 8–12. Bars: 50 µm

RPT revealed that rotenone-treated rats were practically unable to stay on the rotating rod (1.75 ± 1.25 s) in contrast to vehicle-treated animals which ran 68.83 ± 15.37 s (Fig. 1A *t*(22) = 4.34; *p* = 0.002). In OFT, deterioration of motor skills was also reflected by the reduced distance traveled (Fig. 1B *t*(19) = 2.68; *p* = 0.01).

Regarding mood status, OFT revealed that parkinsonian rats spent more time (Fig. 1C, *t*(21) = 2.36; *p* = 0.027) in the periphery suggesting increased level of anxiety. The anhedonia level increased also upon rotenone treatment as rats consumed less sweetened fluid in SPT (Fig. 1D, *t* (22) = 2.34; *p* = 0.029). Increased relative adrenal weight (Fig. 1E, *t*(21) = − 5.22; *p* = 0.0003), reduced relative thymus weight (Fig. 1F, *t* (22) = 2.88; *p* = 0.008) and bodyweight (Fig. 1G, *t*(22) = 6.16; *p* = 0.000005) suggest long-term increased hypothalamus–pituitary–adrenal (HPA) axis activity in rotenone-treated rats.

**Fig. 2:**
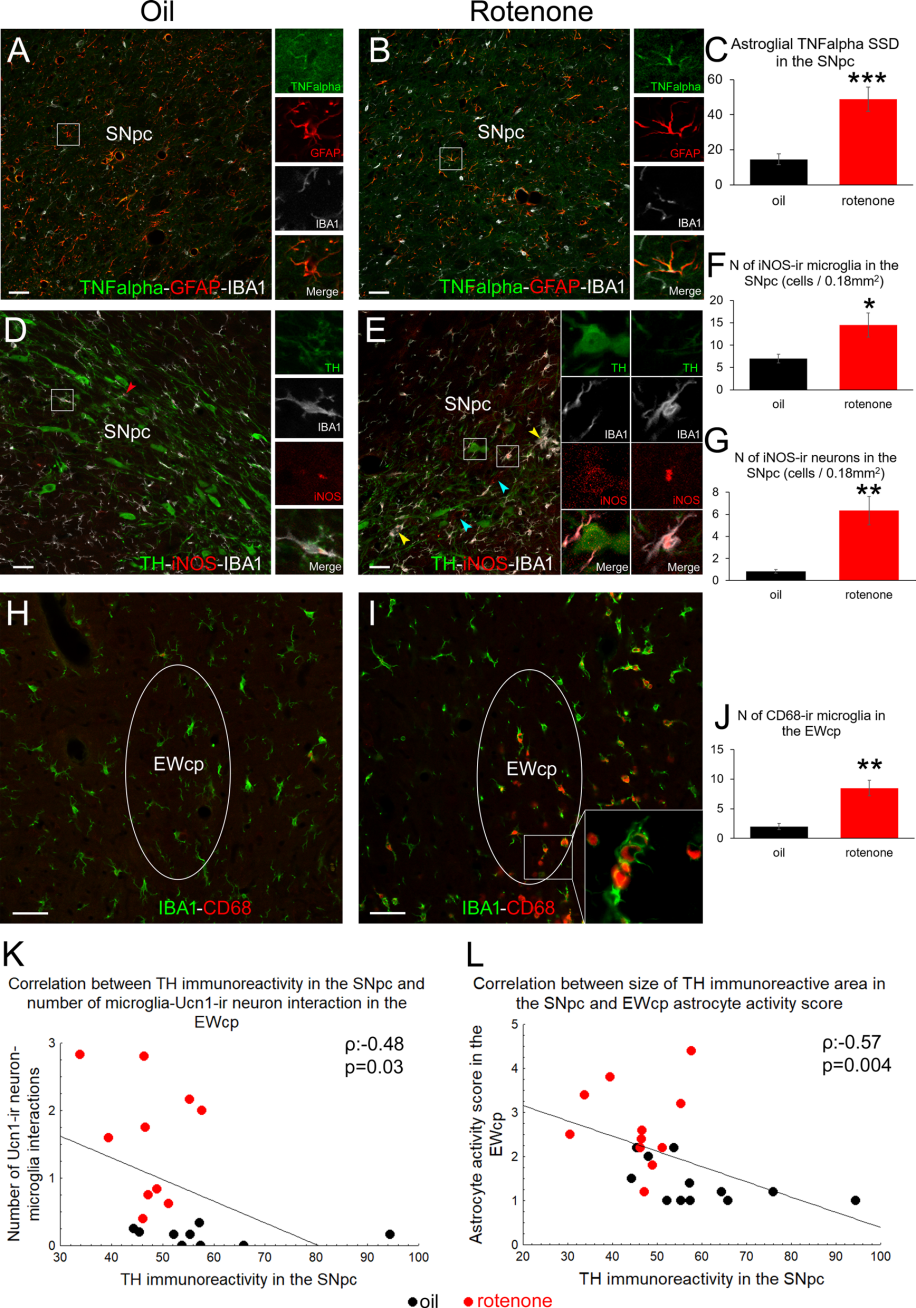
Neuroinflammatory markers support the validity of the rotenone model. Increased tumor necrosis factor alpha (TNF-alpha, green in **A** and **B**) specific signal density (SSD) in glial fibrillary acidic protein (GFAP; red in **A** and **B**)-expressing astrocytes was detected in the substantia nigra pars compacta (SNpc) as shown in graph **C** also. No TNF-alpha immunoreactivity was observed in ionized calcium binding adaptor molecule 1 (IBA1)-containing microglia (white in **A** and **B**). Inducible nitric oxide synthase (iNOS, red in **D** and **E**) immunoreactivity was detected only in very few IBA1 (white in A and B)-containing SNpc microglia in control rats (boxed area and red arrowhead in **D**). Rotenone treatment increased the number of iNOS immunoreactive (-ir) IBA-immunopositive microglia (inset in **E**) that occasionally formed active cell clusters (yellow arrowheads in **E**). Some faintly iNOS-ir nerve cell bodies (blue arrowheads in **E** and graph **G**) were also found and occasionally, TH-ir neurons also appeared to show weak iNOS positivity (see inset in **E**). IBA1 (green in **H** and **I**) and cluster of differentiation 68 (CD68, red in **H** and **I**) double-labeling revealed that upon rotenone treatment (**I**), the microglial cells in the centrally-projecting Edinger–Westphal nucleus (EWcp) co-express CD68 (**J**) suggesting their reactivity. Black bars: vehicle (oil) injected rats, red columns: rotenone-treated group. *p < 0.05, **p < 0.01, ***p < 0.001 in Student’s *t* test, n = 6. Bars: 50 µm. Scatter plot K demonstrates the negative correlation between the TH immunoreactivity in the SNpc and EWcp microglial activity scores. Scatter plot L illustrates the inverse relationship between SNpc/TH immunoreactivity and astroglial activity scores

The number of SNpc/TH-immunoreactive (ir) cells decreased by 32.4% (Fig. 1J, *t* (22) = 6.12; *p* = 0.00004) in rotenone-treated rats. Alpha-synuclein-ir inclusions were found in 10.96 ± 1.58% of SNpc/TH neurons (Fig. 1H, I) in rotenone-treated rats, unlike (0.82 ± 0.42%) in controls (Fig. 1K, *t* (20) = − 6.18; *p* = 0.00005). In addition, IBA1 staining suggested elevated microglial (Fig. 1L–N, *U* (22) = 14; *p* = 0.0008) activation scores, while GFAP revealed increased astroglial activity (Fig. 1O–Q, *U* (22) = 26.5; *p* = 0.008) in rotenone-treated rats. The density of TH-ir nerve fibers was reduced by 69% (Fig. 1T, *t* (19) = 3.41; *p* = 0.003) in the CPu of rotenone-injected rats. Occasionally, also focal loss of striatal TH immunoreactivity was found (Fig. 1R, S). The counting of NeuN-ir cells revealed no neuron loss in the CPu (Fig. 1R, S, U, *t*(19) = 0.57; *p* = 0.57). Furthermore, in the SNpc of rotenone-exposed rats, increased TNF-alpha immunoreactivity in astrocytes (Fig. 2A–C, *t* (22) = − 4.94; *p* = 0.00006) and elevated microglial (Fig. 2D–F, *t*(10) = − 2.62; *p* = 0.025) and neuronal iNOS immunoreactivity (Fig. 2D, E, G, *t*(10) = − 4.25; *p* = 0.0016) was detected, compared to controls.

**Fig. 3:**
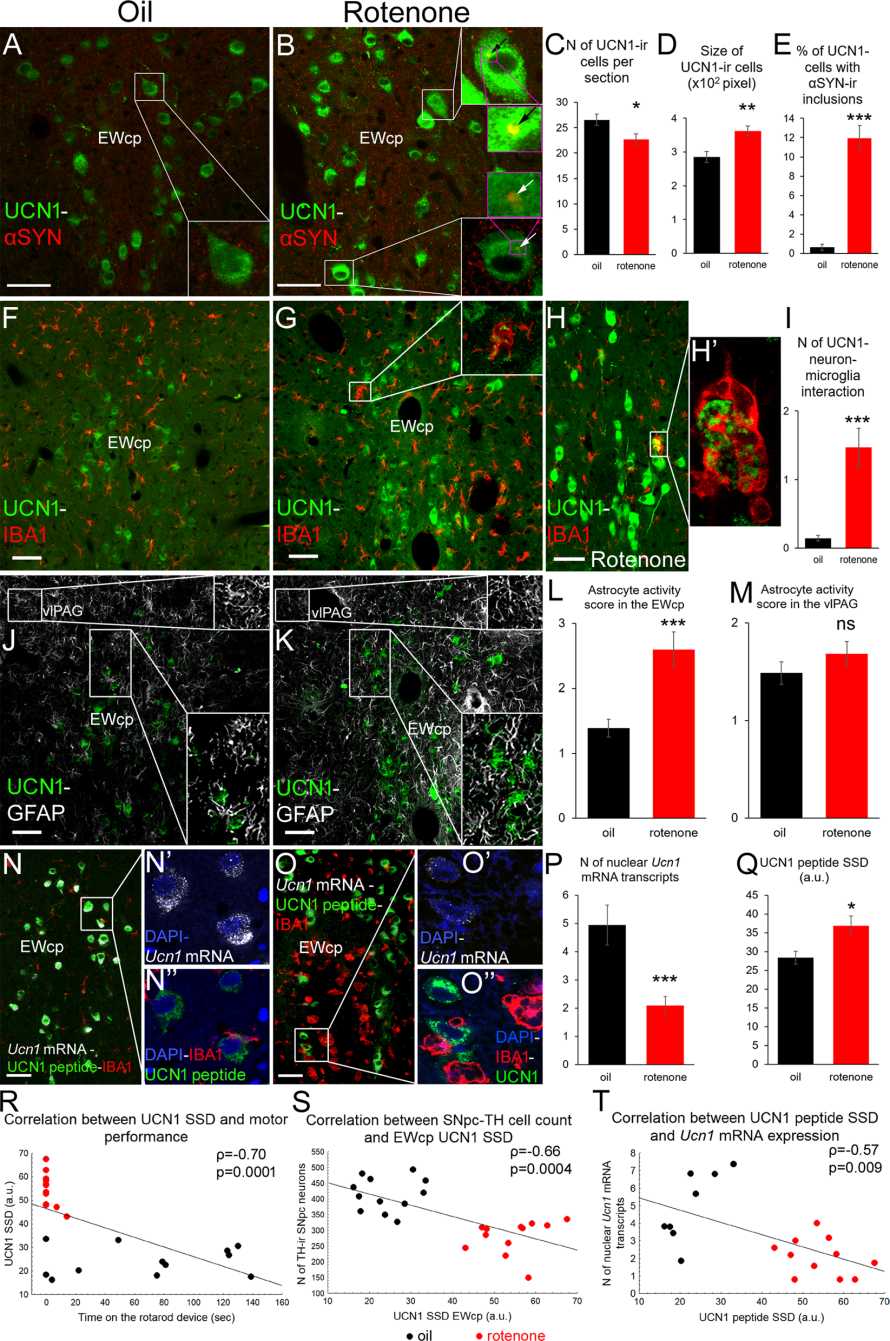
UCN1-immunoreactive (ir) neurons of the centrally-projecting Edinger–Westphal nucleus (EWcp) undergo neurodegeneration and show altered functional neuromorphology in the rotenone model of Parkinson’s disease. In rotenone-treated rats (red bars) the number of EWcp/UCN1 neurons (green in **B**) decreased (**C**), compared with oil-injected controls (**A**, black bars). EWcp/UCN1 neurons also showed swollen morphology (**D**), and contained alpha-synuclein (αSYN, red) immunoreactive (ir) inclusions (see arrows in insets of panel **B** and histogram in **E**). Occasionally, ionized calcium binding adaptor molecule 1 (IBA1)-ir phagocytotic microglial cells (red **G**, **H**) were observed around UCN1 immunoreactive cell fragments (green) (inset in **G**, panels **H** and **H’** and see also Additional file 1: Video 1) and rotenone treatment increased the number of interactions between UCN1 neurons and microglia (**I**). Glial fibrillary acidic protein (GFAP) labeling (white in **J**, **K**) revealed astrogliosis (**L**) upon rotenone treatment in the EWcp, while astrocyte activity score in the adjacent ventrolateral periaqueductal gray (vlPAG) remained unchanged (**M**). RNAscope in situ hybridization for *Ucn1* mRNA (white in **N**, **N’**, **O**, **O’**) revealed reduced expression (**P**) in rotenone-treated rats. Immunolabeling in the same preparations revealed increased UCN1 peptide specific signal density (SSD) of the cells (green in **N**, **N”**, **O**, **O”**) upon rotenone treatment (**Q**). Cells with very low *Ucn1* mRNA and high UCN1 peptide content were occasionally approached by phagocytotic microglial cells (IBA1, red in **O**, **O”**). DAPI **(**4′,6-diamidino-2-phenylindole dihydrochloride) labeling for nuclear staining is shown in blue (**N’**, **N”**, **O’**, **O”**). A negative correlation between UCN1 SSD and motor performance (**R**), SNpc/TH cell count and UCN1 SSD (S) as well as UCN1 SSD and *Ucn1* mRNA expression (**T**) was detected. a.u.: arbitrary unit; *p < 0.05, **p < 0.01, ***p < 0.001 in Student’s *t* test, or in Mann–Whitney *U* test in L and M). *ns* not significant; *n* = 8–12. Bars: 50 µm

When testing our main hypothesis, we found that the number of UCN1 neurons decreased upon rotenone treatment (Fig. 3A–C, t(22) = 2.52; p = 0.019) and they showed pyknotic morphology (Fig. 3A, B, D, *t*(18) = -3.62; p = 0.0019). Occasionally, UCN1 neurons were found to disintegrate, where IBA1-ir microglial processes surrounded the UCN1-ir debris of the neurons (Fig. 3G, H’, Additional file 1: video 1A, Additional file 2: video 1B). Microglia interacted about 10-times more frequently with UCN1 neurons in rotenone-treated rats (Fig. 3I, *t* (18) = − 4.28; *p* = 0.0004) than in controls. Alpha-synuclein-ir cytoplasmic inclusions were observed in 11.94 ± 1.28% of the UCN1 neurons in rotenone-treated rats (Fig. 3B, E), unlike in controls (Fig. 3A, E; 0.62 ± 0.33%; *t*(20) = − 8.52; *p* < 10^–6^). GFAP labeling in the EWcp (Fig. 3J, K) revealed that rotenone induced also astrocyte activation (Fig. 3L; Mann–Whitney test; *U*(22) = − 14.50; *p* = 0.0009). To test whether astrogliosis was restricted to EWcp within the periaqueductal gray matter (PAG), astrocyte morphology was assessed in the ventrolateral PAG (Fig. 3J, K) and no remarkable astrogliosis was seen here (Fig. 3M, *U* (22) = 52.00; *p* = 0.24). Correlation analyses revealed significant inverse relationship of the SNpc/TH immunoreactivity both with microglial (Fig. 2K; Spearman’s *ρ*:-0.48; *p* = 0.03) and with astrocyte activity scores (Fig. 2L; Spearman’s *ρ*: − 0.57; *p* = 0.004) in the EWcp. In contrast, no correlation was found between SNpc/TH immunoreactivity and TNF-alpha (Spearman’s *ρ*: − 0.26; *p* = 0.79) or iNOS (Spearman’s *ρ*: − 0.82; *p* = 0.42) immunoreactivities (data not illustrated).

Rotenone treatment resulted in 126%-elevation of UCN1 peptide immunosignal (compare Fig. 3A with 3B or 3 N” with 3O”) (Fig. 3Q, *t*(22) =  − 11.11; p < 10^–6^). We successfully combined immunofluorescence with RNAscope in situ hybridization for *Ucn1* mRNA. The EWcp perikarya contained high amount of cytoplasmic *Ucn1* mRNA that was often inhomogeneously distributed in clusters of confluent signal dots, especially in rotenone-treated animals. This uneven distribution made the quantitation of cytoplasmic mRNA signal difficult. In contrast, well-countable *Ucn1* mRNA signal puncta were dispersed in the karyoplasm of neurons (Fig. 3N’, O’). Quantitation of intranuclear *Ucn1* mRNA transcripts revealed decreased *Ucn1* mRNA expression by 57.8% in rotenone-treated rats (Fig. 3P; *t*(18) =  − 4.08; *p* = 0.0008). A strong negative correlation was found between cellular UCN1-SSD and nuclear *Ucn1* mRNA content (Fig. 3T, Spearman’s *ρ* = − 0.57; *p* = 0.009). The IBA1 co-labeling identified some EWcp neurons with very low *Ucn1* mRNA content approached by reactive microglial cells showing phagocytotic morphology (Fig. 3O–O”). Similar microglia in other sections were found to co-express CD68 (Fig. 2H–J, *t*(10) =  − 4.53; *p* = 0.001). Correlation analyses also support the recruitment of EWcp in PD, as EWcp/UCN1 content correlated both with the rotarod performance (Fig. 3R, Spearman’s *ρ* = − 0.70; *p* = 0.0001) and SNpc/TH cell counts (Fig. 3S, Spearman’s *ρ* = − 0.66; *p* = 0.004).

**Fig. 4:**
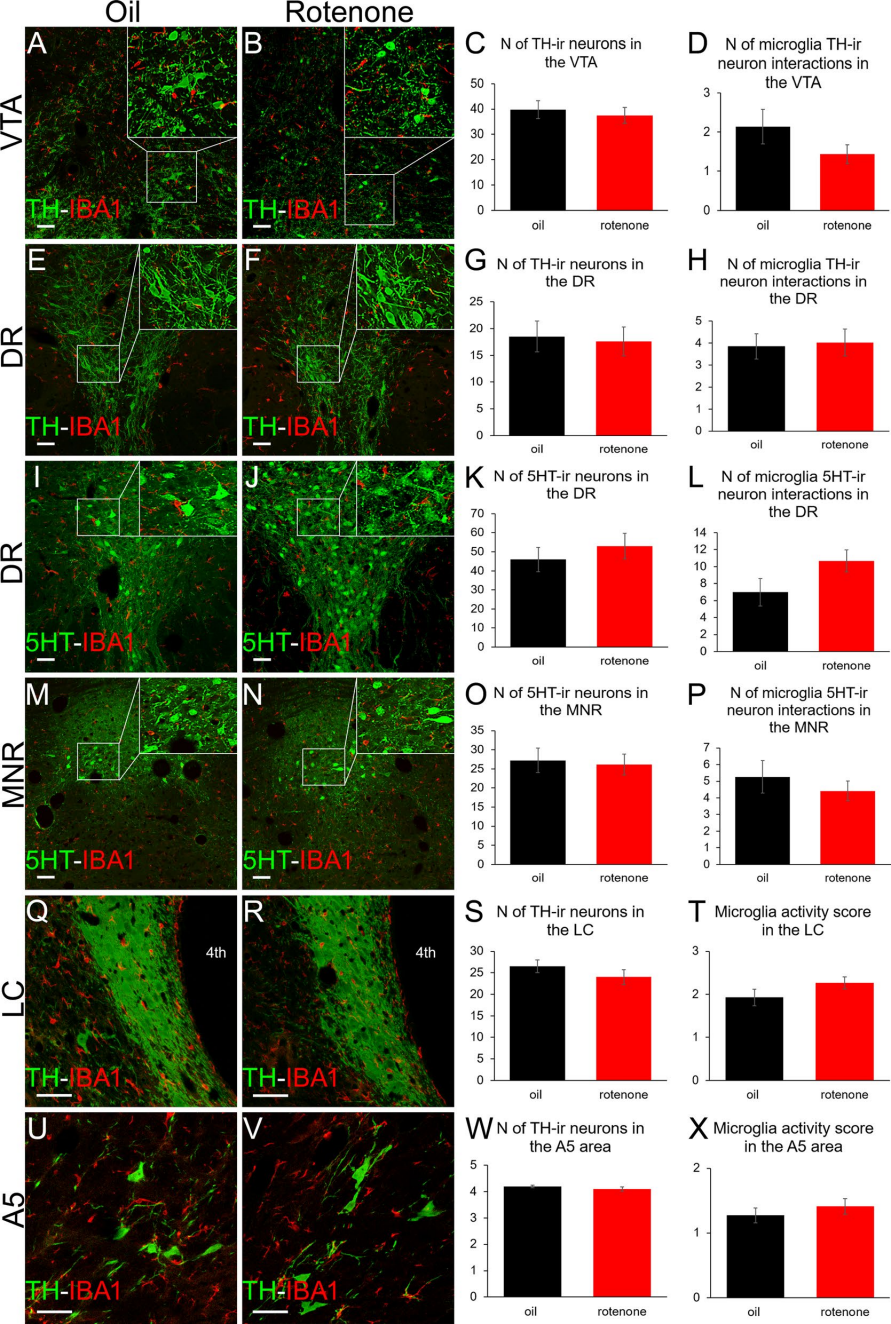
Dopaminergic, serotonergic and noradrenergic centers do not show significant neurodegeneration or glial activation in the rotenone model in the rat. Dopaminergic neurons marked by tyrosine-hydroxylase (TH, green) labeling in the ventral tegmental area (VTA) (**A**, **B**) and dorsal raphe nucleus (DR) (**E**, **F**) do not show significant neurodegeneration as shown by histograms **C** and **G**, respectively. The number of ionized calcium binding adaptor molecule 1 (IBA1, red in **A**, **B**, **E**, **F**)-ir microglia-TH neuron interactions remained unchanged both in the VTA (**D**) and DR (**H**) upon rotenone treatment. The number of serotonin (5-HT)-producing neurons of the DR (green, **I**, **J**) and median raphe nuclei (MNR, green, **M**, **N**) were not affected significantly as shown in panel (**K**) and (**O**), respectively. The number of microglia (IBA1, red in **I**, **J**, **M**, **N**)—5-HT neuron interactions did not change in the DR (**L**) and MNR (**P**). Noradrenergic cells of the locus ceruleus (LC, **Q**, **R**) and A5 noradrenergic cells of the ventrolateral medulla (**U**, **V**) shown by TH immunolabeling (green) did not suffer significant neurodegeneration upon rotenone treatment as shown in histograms (**S**) and (**W**), respectively. The microglial activity score (IBA1, red in **Q**, **R**, **U**, **V**) did not change in the LC (**T**) and in the A5 (**X**). 4th: fourth brain ventricle. Black bars: vehicle (oil) injected rats, red columns: rotenone-treated group. *n* = 7-12. Bars: 50 µm

The toxin distribution in systemic rotenone administration is not restricted to the dopaminergic SNpc and UCN1-ir EWcp. To test if the damage of other mood control-related brain regions contributed to PD-associated depression and anxiety in this model, we also studied dopaminergic, serotonergic and noradrenergic brainstem centers. Neither VTA (Fig. 4A–C, *t*(22) = 0.48; *p* = 0.63) nor DR (Fig. 4E–G, *t*(16) = 0.22; *p* = 0.82) suffered dopaminergic neuron loss. Assessment of TH-ir cell–microglia interactions revealed no significant rotenone effect in the VTA (Fig. 4A, B, D, *t*(21) = 1.30.; *p* = 0.20) and DR (Fig. 4E, F, H, *t*(21) = − 0.21; *p* = 0.83). No serotonergic neuron loss was found in the DR (Fig. 4I–K, t(15) = − 0.73; *p* = 0.47) and MNR (Fig. 4M–O, t(15) = 0.26; p = 0.79). Rotenone treatment did not increase the number of microglia–5HT-ir neuron interactions in DR (Fig. 4, I, J, L, *t*(15) = − 1.76; *p* = 0.099) and MNR (Fig. 4, M, N, P, *t*(15) = 0.78; *p* = 0.44) either. Noradrenergic neuron loss was found neither in the LC (Fig. 4Q–S, t(15) = 1.07; *p* = 0.30) nor in the A5 area (Fig. 4U–W, t(18) = 0.26; *p* = 0.79). No significant microglia activation was seen in the LC (Fig. 4Q, R, T, *U*(19) = 35.00; *p* = 0.45) and A5 (Fig. 4U, V, X, *U*(18) = 24.50; *p* = 0.16).

### Selective EWcp/UCN1 neuron ablation replicates mood status-related symptoms observed in parkinsonian rats

EWcp/UCN1 neurons were selectively ablated by targeted leptin–saporin toxin injection to test whether the administration causes similar behavioral anomalies to those observed in the rotenone model.

**Fig. 5:**
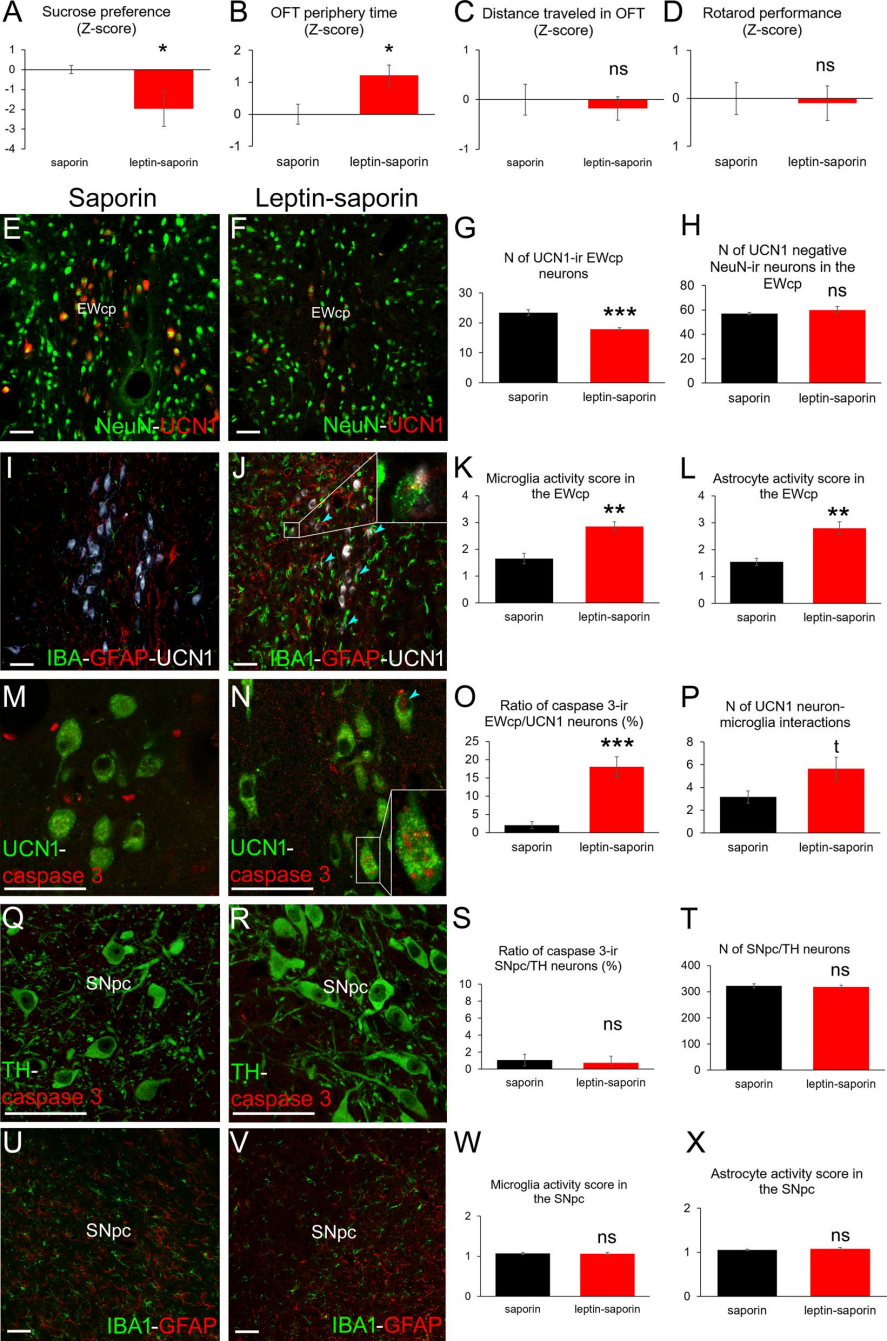
Selective partial UCN1 neuron ablation in the centrally-projecting Edinger-Wespthal nucleus (EWcp) causes increased anhedonia and anxiety level without affecting motor skills. Leptin–saporin-injected rats (red bars) show reduced preference to the sweetened water in the sucrose preference test (**A**). Open field test (OFT) revealed longer period of time spent in the peripheral part of the arena (**B**). Neither the total traveled distance in OFT (**C**) nor the rotarod test (**D**) revealed compromised motor performance of leptin-saporin-treated rats in comparison to saporin-injected controls (black bars). UCN1 (red)—NeuN (green) double labeling (**E**, **F**) revealed reduced UCN1/NeuN cell count upon leptin-saporin treatment (**F**, **G**), while the number of UCN1 immunonegative NeuN neurons remained unchanged in the EWcp (**H**). Ionized calcium binding adaptor molecule 1 (IBA1)-immunoreactive (ir) microglia (green in **I** and **J**) showed increased activity upon leptin-saporin treatment (**J**, **K**) and tended to interact with UCN1-ir cells more frequently (see the blue arrowheads in **J** and graph **P**). Astrocytes showing glial fibrillary acidic protein (GFAP, red in **I** and **J**) were significantly (**L**) more active upon leptin-saporin injection (**J**), although some active cells were also seen in saporin-treated controls (**I**) representing reactive gliosis due to the effect of surgical manipulation. UCN1-ir (green in **M** and **N**) neurons were seen to contain the apoptotic marker caspase 3 (see the boxed area and blue arrowhead in **N**) in leptin-saporin injected rats (**O**), while in saporin-injected control animals, only some glial nuclei were positive for caspase 3 (**M**). Saporin (**Q**) and leptin–saporin injection (**R**) into the EWcp did not affect the number of tyrosine-hydroxylase (TH, green in **Q** and **R**)-ir cells in the substantia nigra pars compacta (SNpc) as revealed by unbiased stereological cell counting (**T**). Caspase 3 (red in **Q** and **R**) revealed no remarkable apoptotic activity of SNpc/TH (**S**). Both microglia (IBA, green in **U** and **V**) and astroglial (GFAP) activity scores remained basal (**W** and **X**, respectively) in both groups (*n* = 6–9) t: tendency (*p* = 0.051), *p < 0.05, **p < 0.01, ***p < 0.001 in Student’s *t* test or in Mann–Whitney *U* test in **K**, **L**, **W**, **X**. *ns* not significant. Bars: 50 µm

SPT revealed that leptin–saporin caused increased anhedonia (Fig. 5A, *t*(16) = 2.13; *p* = 0.048). The UCN1 neuron-ablated rats spent longer time (Fig. 5B, *t*(16) = − 2.70; *p* = 0.015) next to the walls in OFT, suggesting increased anxiety. Importantly, these animals traveled the same total distance as controls (Fig. 5C, *t*(16) = 0.45; *p* = 0.65). RPT also showed that leptin–saporin injection did not affect motor performance (Fig. 5D, *t*(16) =  − 0.20; *p* = 0.84).

UCN1–NeuN labeling proved that leptin–saporin injection reduced the number of EWcp/UCN1 cells by 24.5% (Fig. 5E, G, *t*(16) = 4.56; p = 0.0003). No significant reduction of UCN1 immunonegative but NeuN-ir EWcp cells was seen (Fig. 5E, F, H, *t*(16) = − 1.15; *p* = 0.26), suggesting selective neurodegeneration of EWcp/UCN1 neurons. In contrast to saporin-injected controls, a subset of UCN1 neurons in leptin–saporin treated rats were seen to contain nuclear caspase 3 immunoreactivity suggesting still ongoing apoptotic process (Fig. 5M–O, t(12) =  − 5.52; *p* = 0.0002). Both microglia (Fig. 5I–K *U*(12) = 1.00; *p* = 0.006) and astrocyte (Fig. 5I, J, L, *U*(12) = 0.00; *p* = 0.004) activity scores were higher in the EWcp of leptin–saporin-injected rats than in saporin-treated controls. Microglia in UCN1-ablated rats showed a strong tendency (Fig. 5I, J, P, *t*(10) =  − 2.21; *p* = 0.051) to interact more frequently with UCN1 neurons.

In the SNpc, we did not observe dopaminergic neuron loss (Fig. 5Q, R, T, *t*(10) = 0.41; *p* = 0.68), considerable neuronal caspase 3 activity (Fig. 5Q–S, *t*(10) = 0.79; p = 0.43), micro- (Fig. 5U–W, *U*(12) = 13.00; *p* = 0.42) and astrogliosis (Fig. 5U–W, X, *U*(12) = 14,50; *p* = 0.57) in the leptin–saporin model.

## Discussion

In the systemic toxic model of Parkinson’s disease, rotenone was chosen to assess whether the PD-like state can be associated with morphological changes also within the peptidergic EWcp and whether it is accompanied with depression-like phenotype and anxiety. Next, a local neuron ablation was performed to examine whether the selective EWcp-damage causes comparable deterioration in the mood status.

### Anxiety and depression as non-motor symptoms of PD are detected in the rotenone model

Although not without limitations [41, 42], the rotenone model of PD reproduces many features of the human disease, including systemic mitochondrial impairment, selective dopaminergic nigrostriatal damage, microglia activation, alpha-synuclein accumulation with formation of LBs [43, 44]. The RPT, a widely used method [45, 46] unambiguously revealed severe motor coordination deficit in rotenone-treated rats. Reduced OFT locomotor activity further supported the deterioration of motor skills in full agreement with earlier studies [47, 48]. OFT also suggested increased anxiety in rotenone-treated rats [49]. In SPT, reduced sucrose consumption in parkinsonian rats suggested diminished reward-seeking behavior [50]. Although these in vivo results confirmed the efficacy of the PD model, one has to consider the limitations of these tests as motor skills of rats might directly affect their performance. Nevertheless, after pre-testing other mood-status assessing paradigms (e.g., forced swim test, light–dark box test) in our pilot studies, we decided for OFT and SPT as parkinsonian animals were also capable of performing these, and the within and between subject error was acceptable.

The number of SNpc/TH cells was reduced by 32.4% in rotenone-treated rats. Although this is considerably less than the 70% neuron loss in humans [1], rats with 20% loss of SNpc/TH neurons showed significantly impaired motor skills. Furthermore, in line with others, we found neurons with cytoplasmic alpha-synuclein inclusions in SNpc/TH [51] that correspond to LB-like structures. These two observations strongly suggest the validity of our model. Increased SNpc/astroglia TNF-alpha immunoreactivity and elevated microglial and neuronal iNOS signal in the SNpc strongly suggest that the rotenone treatment-related oxidative stress and consequent neuroinflammation contributed to the neuron loss [52, 53]. Furthermore, in the rotenone model a diffuse striatal axonal loss and also drastic focal TH-ir fiber loss may be observed [54]. Indeed, we also measured reduced CPu/TH-ir fiber content in rotenone-treated rats, and found some circumscribed striatal neuropil areas with almost undetectable TH-ir (Fig. 1S).

### EWcp/UCN1 neurons suffer neurodegeneration and surviving cells show functional damage in the rotenone model

The magnitude of neuronal loss, the presence of cytoplasmic alpha-synuclein-ir inclusions, corresponding to LB-like structures and the increased microglial activity in the SNpc was in line and in statistical correlation with micro- and astroglial activity as well as with UCN1–neuron loss of the EWcp suggesting their similar susceptibility to the toxic agent. This is not surprising as EWcp neurons are developmentally also closely related with SNpc/TH neurons [55]. Although obvious cytoplasmic alpha-synuclein immunoreactive inclusions suggested the presence of LB-like structures in the SNpc and EWcp, we have to state an important limitation here is that our antibody, similarly to numerous other commercially available sera [56], was not specific for the high molecular weight isoforms found in LBs in the rat.

Beyond the obvious interaction between UCN1-ir cell fragments (Fig. 3H’) and CD68-ir reactive microglia (Fig. 2I) [57] that unambiguously proves the loss of EWcp/UCN1 cells, we also provide evidence that surviving UCN1 cells suffered functional damage. We assume that the mitochondrial complex I inhibition in UCN1 cells caused energy deficit [58] with consequent UCN1 peptide accumulation due to impaired axonal transport. The increased SSD of UCN1 suggests peptide accumulation in the perikarya of cells, which was also observed in stressed mice [19, 59]. The reduction of *Ucn1* mRNA message is in line with findings in EWcp samples of chronically stressed rats [23] and depressed suicide victims [25]. Adrenal-, thymus- and bodyweight data suggest increased HPA axis activity that is commonly associated with depression-like states in animal models [19, 60] and human depression [17].

Although PD-related neurodegeneration was shown to affect the DR [3], VTA and LC [5], we did not observe remarkable damage of these serotonergic, dopaminergic and noradrenergic cell populations in our rats. We cannot exclude that a higher rotenone dose [30] or a longer treatment period would have also affected these or other centers [53], that were not examined in this project but may affect mood status. Nevertheless, our rat model ultimately supports further the significance of the EWcp/UCN1 in PD-associated mood disorder.

### Local targeted toxic UCN1–neuron ablation results in altered mood status

To further corroborate that EWcp/UCN1 neuron loss contributes to deterioration in the mood status, a local targeted UCN1 cell ablation was conducted. The leptin-conjugated saporin is internalized into leptin receptor positive cells leading to disturbed protein synthesis [61] and apoptotic neuron loss [62]. As in this area the leptin receptor expression is restricted to a part of EWcp/UCN1 cells [10, 63], a selective partial UCN1 neuron death was expected. After pre-tests with multiple leptin–saporin doses we achieved a cell loss that was comparable with that observed in the rotenone model. Earlier works [33] and pre-tests suggested that the cell death requires 2 weeks in the leptin–saporin model. Nevertheless, the occasional caspase 3 immunoreactivity in UCN1 neurons suggested that in some cells the leptin–saporin-induced apoptotic process [64] was still in progress at the time of tissue sampling. Some activated micro- and astroglial cells were seen also in saporin-injected controls most likely due to the direct physical effect of the surgery. In leptin–saporin-treated rats both glial cell types were more reactive and microglia tended to interact more frequently with the UCN1 neurons. Nevertheless, the selectivity of ablation was confirmed by the unchanged number of UCN1 immunonegative, but NeuN positive EWcp cells upon leptin–saporin treatment. In leptin–saporin-injected animals, increased OFT-anxiety and SPT–anhedonia suggested that the loss of EWcp/UCN1 cells provoked mood changes but normal motor coordination and locomotor activity excluded parkinsonism. In line with this, dopaminergic neuron loss, apoptotic activity or reactive gliosis was not detected in the SNpc.

## Conclusion

In this study we successfully applied the rotenone model of PD and detected the anxious and depression-like phenotype, considered as non-motor symptoms of PD in the rat. Selective local UCN1 neuron ablation evoked similar mood status without motor symptoms. With respect to the limitations, our findings collectively suggest that the impairment of the UCN1 neurons in the EWcp contribute to the non-motor symptoms of PD. Human studies have to determine the potential diagnostic and therapeutic significance of these observations.

## Supplementary Information

**Additional file 1: Video 1A ** shows a 3D reconstruction of confocal Z-stack images demonstrating a disintegrating UCN1 neuron (green) surrounded by phagocytotic IBA1 immunoreactive microglia (red) cells in the EWcp. (See the same cell in Fig. 3 also.).

**Additional file 2: Video 1B.** shows a  3D reconstruction of confocal Z-stack images demonstrating a disintegrating UCN1 neuron (green) surrounded by phagocytotic IBA1 immunoreactive microglia (red) cells in the EWcp. (See the same cell in Fig. 3 also.).

## Data Availability

The data sets used and/or analyzed during the current study are available from the corresponding author on reasonable request.

## Abbreviations

5-HT: Serotonin
CD68: Cluster of differentiation 68
CPu: Caudate–putamen
CRH: Corticotropin-releasing hormone
DAPI: 4′,6-Diamidino-2-phenylindole
DR: Dorsal raphe nucleus
EW: Edinger–Westphal nucleus
EWcp: Centrally-projecting Edinger–Westphal nucleus
GFAP: Glial fibrillary acidic protein
HPA: Hypothalamus–pituitary–adrenal axis
IBA1: Ionized calcium-binding adapter molecule 1
iNOS: Inducible nitric oxide-synthase
LB: Lewy body
LC: Locus ceruleus
MNR: Median raphe nucleus
OFT: Open field test
PAG: Periaqueductal gray matter
PD: Parkinson’s disease
RPT: Rotarod performance test
SNpc: Substantia nigra, pars compacta
SPT: Sucrose preference test
SSD: Specific signal density
TH: Tyrosine-hydroxylase
TNF-alpha: Tumor necrosis factor alpha
UCN1: Urocortin 1 peptide
*Ucn1*: Urocortin 1 mRNA
VTA: Ventral tegmental area
